# Association of triglyceride-glucose index with myocardial injury post-stroke in older patients with first-ever ischemic stroke

**DOI:** 10.1186/s12877-023-04041-7

**Published:** 2023-06-08

**Authors:** Mu Niu, Zhikang Zhou, Long Wang, Jian Yang, Miao Sun, Xin Lv, Faqiang Zhang

**Affiliations:** 1 Department of Neurology, The Affiliated Hospital of Xuzhou Medical University, Xuzhou Medical University, Xuzhou, 221002 Jiangsu China; 2 Department of Anesthesiology, The First Medical Center, Chinese PLA General Hospital, Beijing, 100853 China; 3 Department of Pain Medicine, The First Medical Center, Chinese PLA General Hospital, Beijing, 100853 China; 4 Department of Anesthesiology, Shanghai Pulmonary Hospital, Tongji University School of Medicine, Shanghai, 200433 China

**Keywords:** Triglyceride-glucose index (TyG index), Myocardial injury post-stroke, Insulin resistance, Older patients, Biomarker

## Abstract

**Background:**

Myocardial injury post-stroke is a common sequela of acute stroke. Triglyceride-glucose index (TyG index), a valuable surrogate indicator of insulin resistance, has been suggested to be closely related to cardiovascular outcomes. However, it is unknown whether the TyG index is independently associated with a higher risk of myocardial injury post-stroke. We therefore investigated the longitudinal association between TyG index and risk of myocardial injury post-stroke in older patients with first-ever ischemic stroke and no prior cardiovascular comorbidities.

**Methods:**

We included older patients with first-ever ischemic stroke and no prior cardiovascular comorbidities between January 2021 to December 2021. The individuals were stratified into low and high TyG index groups according to the optimal cutoff value with TyG index. We performed logistic regression analysis, propensity score matching (PSM) analysis, restricted cubic spline analysis, and subgroup analyses to explore the longitudinal association between TyG index and risk of myocardial injury post-stroke.

**Results:**

We included 386 individuals with a median age of 69.8 years (interquartile range: 66.6, 75.3). The optimal TyG index cut-off for predicting myocardial injury post-stroke was 8.9 (sensitivity 67.8%; specificity 75.5%; area under curve 0.701). Multivariate logistic regression analysis revealed that the risk of genesis of myocardial injury post-stroke increased with elevated TyG index (odds ratio [OR], 2.333; 95% confidence interval [CI], 1.201–4.585; *P* = 0.013). Furthermore, all covariates were well balanced between the two groups. The longitudinal association between TyG index and myocardial injury post-stroke remained significantly robust (OR: 2.196; 95% CI: 1.416–3.478; *P* < 0.001) after PSM adjustment.

**Conclusion:**

Individuals with an elevated TyG index were more susceptible to having an increased risk of myocardial injury post-stroke. TyG index thus might be served as a complementary approach for optimized-for-risk stratification in older patients with first-ever ischemic stroke and no prior cardiovascular comorbidities.

**Supplementary Information:**

The online version contains supplementary material available at 10.1186/s12877-023-04041-7.

## Introduction

Cardiac complications represent a formidable challenge after acute stroke [1, 2]. Over 1.5 million deaths worldwide are attributed to neurocardiogenic syndromes, including myocardial injury post-stroke, acute coronary syndromes, left ventricular dysfunction, cardiac arrhythmia, and neurogenic sudden cardiac death [2–4]. Cardiac troponin (cTn), a cardiac biomarker of myocardial injury, is often elevated after stroke [5]. Due to the cTn elevation without typical signs or symptoms, such as chest pain or dyspnea, myocardial injury post-stroke is not appreciated in the clinical setting [6, 7]. Nevertheless, evidence suggests that stroke-induced myocardial injury can contribute to an augmented risk of mortality and severe cardiovascular complications [8–10]. Therefore, identifying new modifiable risk factors for stroke-induced myocardial injury will help to characterize high-risk patients in the early stage and improve therapeutic efficacy.

Insulin resistance is characterized by the reduced sensitivity of target organs to insulin and the consequent dysregulation in glucose uptake [11, 12]. Insulin resistance can contribute to numerous metabolic disturbances including hyperglycemia, hypertension, and hyperlipidemia, which may predispose individuals to develop cardiovascular and cerebrovascular diseases [13–15]. Thus, insulin resistance is not only a causative risk factor but also a predictor of cardiovascular and cerebrovascular disorders. Therefore, it will be of immediate clinical significance, and urgent need to develop screening tools to evaluate insulin resistance and improve diagnostic precision. Triglyceride-glucose (TyG) index, derived from fasting triglyceride and fasting plasma glucose, has been proposed to be superior to the homeostasis model assessment of insulin resistance (HOMA-IR) of estimating insulin resistance [16, 17]. Recent studies have documented that individuals with higher TyG index are more susceptible to having an increased risk of major adverse cardiovascular events and cardiovascular diseases [18, 19]. To date, the association between TyG index and stroke-induced myocardial injury remains unidentified among older individuals with first-ever ischemic stroke.

Therefore, we designed and conducted the current study to explore whether the elevated TyG index is intimately linked to the higher risk of stroke-induced myocardial injury in older patients with first-ever ischemic stroke and no prior cardiovascular diseases.

## Methods

This study was approved by the Medical Ethics Committee of The Affiliated Hospital of Xuzhou Medical University (No.XYFY2021-KL112-01). This study strictly adhered to the applicable guidelines of the Declaration of Helsinki. Given the retrospective nature of the cohort study, the need for informed content was waived. All data were anonymized before analysis.

### Study population

In this retrospective cohort study, we included adults ≥ 65 years old with acute ischemic stroke who were admitted to the Affiliated Hospital of Xuzhou Medical University from January 2021 to December 2021. We excluded patients with (1) prior history of stroke or transient ischemic attack of any type, (2) previously confirmed diagnosis of cancer, and (3) missing data for any confounder. Individuals were excluded if they had prior cardiovascular diseases or cardiac surgeries, including coronary artery disease, valvular heart disease, congestive heart failure, myocardial infarction, atrial fibrillation, percutaneous coronary intervention, coronary artery bypass graft, valve repair or replacement, and any other severe cardiovascular disease.

### Data collection and definitions

Demographic and clinical data were extracted independently from the electronic medical records by trained investigators blinded to the study design. The data included age, sex, body mass index (BMI), systolic and diastolic blood pressure (SBP and DBP), smoking history, history of alcohol abuse, hypertension, diabetes mellitus, peripheral vascular disease, renal dysfunction, and medications before admission (antihypertensive agents, lipid-lowering medications, antidiabetic agents or insulin). Stroke laterality, stroke location, stroke severity (national institutes of health stroke scale, NIHSS), and thrombolysis during hospitalization were also noted. Fasting venous blood samples were collected within 24 h after hospital admission. Peripheral platelet, neutrophil, lymphocyte, hemoglobin, albumin, fasting plasma glucose (FBG), hemoglobin A1c (HbA1c), total cholesterol (TC), triglyceride (TG), high-density lipoprotein cholesterol (HDL-C), low-density lipoprotein cholesterol (LDL-C), and uric acid were analyzed. Additionally, neutrophil-to-lymphocyte ratio (NLR), and platelet-to-lymphocyte ratio (PLR) were calculated as follows: NLR = neutrophil/lymphocyte, PLR = platelet/lymphocyte. TyG index was calculated as ln [fasting triglyceride (mg/dl) × fasting plasma glucose (mg/dl)/2] [20].

Blood samples for up-to-date generation 5 (high-sensitivity) troponin T or I were collected on the first 2–3 days after stroke while patients were in hospital [21]. Additionally, medical data including electrocardiogram changes and ischemic symptoms (ie, chest pain or radiation to the arm, neck, black or jaw, and shortness of breath or dyspnea) were also obtained by reviewing electronic medical records.

### Clinical outcome

The primary outcome of interest was incident myocardial injury post-stroke. Myocardial injury was defined when the elevation of available cTn concentrations exceeded the generation-specific or assay-specific 99th percentile upper reference limit and was apparently of ischemic origin during hospitalization, with or without clinical symptoms or signs [21, 22].

### Statistical analysis

Continuous data were tested for normality and homogeneity of variances before performing statistical tests. The data were expressed as mean ± standard deviation (SD), or median with interquartile range (IQR), as appropriate. Categorical data were presented as numbers and percentages. Receiver operating characteristic (ROC) curve analysis was performed to determine the optimal cutoff value with the highest Youden index for high or low distribution of TyG index. The area under curve was performed to evaluate the predictive value of TyG index for incident myocardial injury post-stroke. TyG index was also assessed by continuous and quartile variables. Multivariate logistic regression model analyses based on stepwise method were performed to investigate the association between TyG index and incident myocardial injury post-stroke. To adjust the potential confounders, we constructed five univariate or multivariate models to calculate the odds ratio (OR) of TyG index and myocardial injury post-stroke. Model 1 was an unadjusted univariate model. Model 2 was adjusted for age, sex, BMI, SBP, DBP, smoking history, history of alcohol abuse, hypertension, diabetes mellitus, peripheral vascular disease, renal dysfunction, and medications before admission including antihypertensive agents, lipid-lowering medications, antidiabetic agents or insulin. Model 3 was adjusted for stroke laterality, stroke location, stroke severity, and thrombolysis during hospitalization. Model 4 included clinical laboratory data such as hemoglobin, albumin, FBG, HbA1c, TC, TG, HDL-C, LDL-C, uric acid, NLR, and PLR. Model 5 was adjusted for all the potential confounders as the full regression model. Dose–response relationship between the TyG index and incident myocardial injury post-stroke was further evaluated using restricted cubic spline analysis, with two knots at the 25th and 75th percentiles and the reference point at the median of TyG index.

Additionally, to account for the imbalance between groups in baseline characteristics, we performed propensity score matching (PSM) to generate a new cohort in which patients with high or low TyG index would achieve balances on key variables. A propensity score of different levels of TyG index was computed with the multivariate logistic regression model, in which TyG index was the outcome variable whereas the other baseline parameters were independent variables. The baseline parameters were utilized during the PSM process, including age, sex, BMI, SBP, DBP, cigarette-smoking, alcohol, hypertension, diabetes mellitus, peripheral vascular disease, renal dysfunction, antihypertensive agents, lipid-lowering agents, antidiabetic agents or insulin, stroke laterality, stroke location, stroke severity (NIHSS), thrombolysis, hemoglobin, albumin, NLR, PLR, HbA1c. Patients with high or low TyG index were randomly assigned and matched (1:1) using the greedy nearest-neighbor matching approach within maximum caliper width of 0.2. We applied kernel density plots to estimate the distributions of propensity scores between groups. Standardized mean difference (SMD) was performed for the comparison between groups at baseline, where a value < 0.1 indicated as minor acceptable deviation [23].

Male, hypertension, diabetes mellitus, and stroke severity were associated with an increased risk of incident myocardial injury [24, 25]. Thus, we fit subgroup analyses to account for the effect of TyG index on the myocardial injury post-stroke according to sex, hypertension, diabetes mellitus, and stroke severity.

Statistical significance was considered for a two-sided *P* < 0.05. IBM SPSS Statistics (version 26.0, IBM, Corp.) and R statistical software (R version 4.0.5, R Foundation for Statistical Computing) were used in all statistical analyses.

## Results

### Study population

**Fig. 1:**
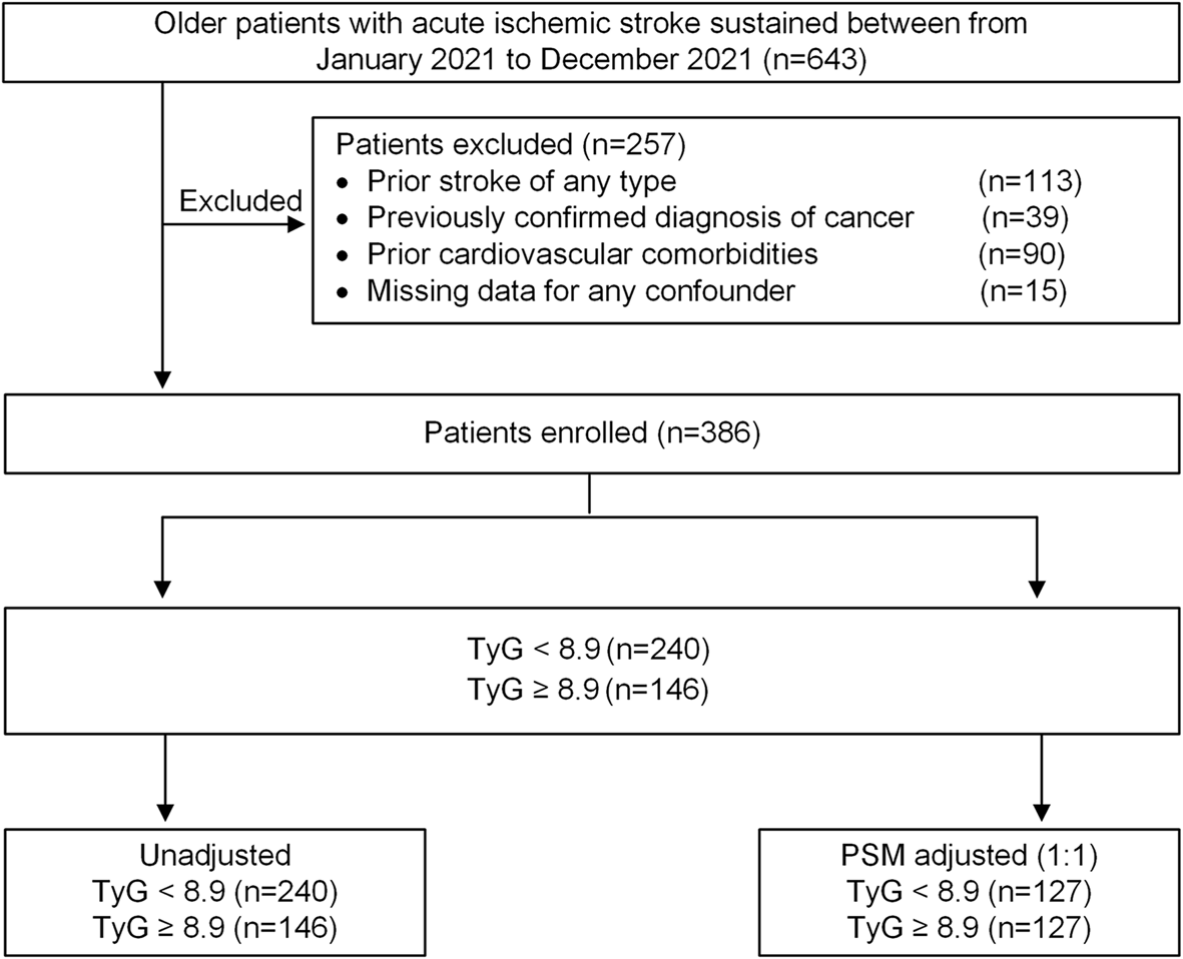
Study profile. TyG index, triglyceride-glucose index; PSM, propensity score matching

A total of 643 older patients underwent acute ischemic stroke between January 2021 and December 2021. After applying a series of exclusion criteria (Fig. 1), 386 older individuals with first-ever ischemic stroke and no prior cardiovascular comorbidities were enrolled in the final analysis, with a median age of 69.8 years (IQR: 66.6, 75.3), median BMI 24.8 kg/m^2^ (IQR: 22.8, 27.1), of whom 172 (44.6%) were female. Among those admissions, 199 (51.6%) had cortical infarcts, 64 (16.6%) underwent subcortical lesions, and 113 (29.3%) had multiple stroke lesions. In this cohort, the median NIHSS score at admission was 5.6 (IQR: 2.9, 8.7). In our study population, 133 (34.5%) patients were subjected to myocardial injury within 30 days after stroke. The overall incidence of myocardial injury post-stroke was aligned with prior rates of 30%–60% in stroke patients using up-to-date high-sensitivity assays [22, 26].

The median TyG index was 8.7 (IQR: 8.3, 9.2). Result of the ROC analysis revealed that the optimal cutoff value with TyG index for accurately predicting myocardial injury post-stroke was set at 8.9 (sensitivity 67.8% and specificity 75.5%), with an area under curve of 0.701 (95% CI: 0.673–0.758), and Youden index of 0.229 (Supplementary Fig. 1). Additionally, although its predictive ability was, at best, modest, the TyG index outperformed other predictors such as diabetes mellitus, hypertension, and stroke severity (NIHSS > 4 vs NIHSS ≤ 4) (*P* < 0.05 for all) (Supplementary Table 1 and Supplementary Fig. 2). The individuals were then stratified into two groups according to the optimal cutoff value with TyG index: low group (TyG index < 8.9, *n* = 240, 62.2%) and high group (TyG index ≥ 8.9, *n* = 146, 37.8%). Comparisons of baseline characteristics between the two groups are shown in Table 1. Some baseline demographic and clinical characteristics, such as BMI, hypertension, diabetes mellitus, use of medications at admission including antihypertensive agents and antidiabetic agents or insulin, stroke severity (NIHSS score), HbA1c, fasting plasma glucose, TC, TG, HDL-C and LDL-C, differed between the TyG < 8.9 and TyG ≥ 8.9 groups, and other variables were relatively similar. Patients with high TyG index had more high-risk factors for cardiovascular and cerebrovascular diseases (higher BMI, hypertension, diabetes mellitus, higher long-term treatment of antihypertensive agents and antidiabetic agents or insulin, higher values of TC, TG, and LDL-C, and lower values of HDL-C, than did those with TyG index < 8.9.

**Table 1:** Subject baseline characteristics by binary classification of TyG index

Characteristic	Unadjusted Sample (*n* = 386)				PSM adjusted (1:1) (*n* = 254)			
	TyG < 8.9 (*n* = 240)	TyG ≥ 8.9 (*n* = 146)	*P* value	SMD	TyG < 8.9 (*n* = 127)	TyG ≥ 8.9 (*n* = 127)	*P* value	SMD
TyG index, unit	8.4 (8.1,8.6)	9.3 (9.1,9.6)	< 0.001	0.835	8.4 (8.2,8.6)	9.2 (9.1,9.5)	< 0.001	0.870
Myocardial injury post-stroke (%)	68 (28.3)	65 (44.5)	0.001	0.492	34 (26.8)	53 (41.7)	0.012	0.236
Demographics								
Age, y^b^	69.0 (66.7,75.7)	70.1 (67.0,75.0)	0.156	0.153	69.8 (66.9,74.6)	69.9 (66.8,74.6)	0.562	0.021
Female (%)^b^	101 (42.1)	71 (48.6)	0.209	0.132	55 (43.3)	56 (44.1)	0.899	0.023
BMI, kg/m^2^^b^	24.4 (22.4,26.8)	25.6 (23.5,27.6)	0.003	0.317	24.8 (23.4,27.1)	25.4 (23.4,27.4)	0.459	0.045
SBP, mmHg^b^	134.0 (122.0,147.0)	137.5 (125.3,148.0)	0.123	0.161	138.0 (128.0,149.0)	137.0 (125.0,146.5)	0.495	0.048
DBP, mmHg^b^	80.0 (72.0,86.0)	80.0 (74.0,89.0)	0.177	0.138	81.0 (74.50,87.0)	80.0 (73.00,89.0)	0.525	0.056
Cigarette-smoking (%)^b^	82 (34.2)	43 (29.5)	0.337	0.101	43 (33.9)	40 (31.5)	0.688	0.073
Alcohol (%)^b^	63 (26.3)	45 (30.8)	0.332	0.101	32 (25.2)	31 (24.4)	0.884	0.057
Previous medical history								
Hypertension (%)^b^	112 (46.7)	90 (61.6)	0.004	0.304	78 (61.4)	76 (59.8)	0.797	0.032
Diabetes mellitus (%)^b^	49 (20.4)	61 (41.8)	< 0.001	0.474	42 (33.1)	43 (33.9)	0.894	0.017
Peripheral vascular disease (%)^b^	49 (20.4)	31 (21.2)	0.848	0.020	31 (24.4)	28 (22.0)	0.656	0.089
Renal dysfunction (%)^b^ ^a^	5 (2.1)	4 (2.7)	0.679	0.043	3 (2.4)	2 (1.6)	0.651	0.057
Antihypertensive agents (%)^b^	66 (27.5)	60 (41.1)	0.006	0.273	50 (39.4)	49 (38.6)	0.898	0.016
Lipid-lowering agents (%)^b^	26 (10.8)	14 (9.6)	0.697	0.041	11 (8.6)	12 (9.4)	0.827	0.027
Antidiabetic agents or insulin (%)^b^	43 (17.9)	41 (28.1)	0.019	0.475	36 (28.3)	35 (27.6)	0.889	0.016
Stroke laterality (%)^b^								
Left	94 (39.2)	49 (33.6)	0.199	0.103	42 (33.1)	39 (30.7)	0.920	0.008
Right	90 (37.5)	51 (34.9)			46 (36.2)	48 (37.8)		
Bilateral	56 (23.3)	46 (31.5)			39 (30.7)	40 (31.5)		
Stroke location (%)^b^								
Cortical	128 (53.3)	71 (48.6)	0.889	0.103	65 (51.2)	64 (50.4)	0.903	0.089
Subcortical	39 (16.3)	25 (17.1)			21 (16.5)	20 (15.7)		
Cerebellar	4 (1.7)	2 (1.4)			2 (1.6)	2 (1.6)		
Brainstem	2 (0.8)	2 (1.4)			0 (0.0)	1 (0.8)		
Multiple	67 (27.9)	46 (31.5)			39 (30.7)	40 (31.5)		
Stroke severity (NIHSS), unit^b^	4.0 (1.0,7.0)	6.0 (3.0,8.0)	< 0.001	0.456	5.0 (3.0,8.0)	5.0 (2.0,8.0)	0.112	0.078
Thrombolysis (%)^b^	17 (7.1)	15 (10.3)	0.270	0.114	11 (8.6)	12 (9.4)	0.827	0.043
Laboratory findings								
Hemoglobin, g/L^b^	131.0 (121.0,143.0)	135.5 (121.0,147.0)	0.326	0.101	131.0 (121.0,144.5)	132.5 (121.0,147.0)	0.466	0.039
Albumin, g/L^b^	40.1 (37.5,42.7)	40.9 (38.0,43.0)	0.945	0.007	40.0 (37.0,42.7)	41.0 (38.0,43.0)	0.167	0.078
NLR, unit^b^	2.2 (1.6,3.7)	2.4 (1.8,4.2)	0.086	0.170	2.4 (1.8,3.8)	2.4 (1.8,4.1)	0.640	0.026
PLR, unit^b^	131.3 (93.6,172.4)	132.6 (101.2,172.8)	0.948	0.007	134.0 (94.6,169.0)	132.5 (101.2,172.6)	0.744	0.075
HbA1c, %^b^	5.2 (4.3,6.7)	5.8 (4.9,7.7)	0.034	0.876	5.5 (4.6,6.9)	5.6 (4.8,7.2)	0.157	0.098
FBG, mmol/L	5.0 (4.7,5.8)	6.6 (5.4,8.4)	< 0.001	0.909	5.3 (4.8,6.1)	5.6 (5.3,8.1)	0.023	0.158
TC, mmol/L	4.0 (3.6,5.3)	5.0 (4.4,5.8)	< 0.001	0.922	4.5 (4.1,5.1)	4.9 (4.4,5.5)	0.028	0.175
TG, mmol/L	1.3 (0.8,2.2)	2.1 (1.7,2.7)	< 0.001	0.886	1.6 (1.1,2.0)	2.0 (1.6,2.6)	0.018	0.245
HDL-C, mmol/L	1.2 (1.0,1.4)	0.9 (0.8,1.2)	< 0.001	0.864	1.1 (0.9,1.3)	1.0 (0.9,1.2)	0.052	0.111
LDL-C, mmol/L	2.5 (2.2,3.6)	3.2 (2.7,3.7)	< 0.001	0.767	2.9 (2.4,3.5)	3.3 (2.7,3.8)	0.022	0.189
Uric acid, mmol/L^b^	270.6 (229.4,328.0)	289.8 (234.3,358.1)	0.074	0.184	273.6 (229.4,344.7)	287.6 (225.7,355.9)	0.723	0.048

The data are presented as the median (interquartile range), mean (standard deviation), or n (%)

*Abbreviations*: *TyG index* Triglyceride-glucose index, *PSM* Propensity score matching, *SMD* Standardized mean difference, *BMI* Body mass index, *SBP* Systolic blood pressure, *DBP* Diastolic blood pressure, *NIHSS* National institutes of health stroke scale, *NLR* Neutrophil-to-lymphocyte ratio, *PLR* Platelet-to-lymphocyte ratio, *FBG* Fasting plasma glucose, *HbA1c* Hemoglobin A1c, *TC* Total cholesterol, *TG* Triglyceride, *HDL-C* High-density lipoprotein cholesterol, *LDL-C* Low-density lipoprotein cholesterol

^a^Creatinine > 177 μmol/L

^b^Variables included in the propensity score

### Association between TyG index and myocardial injury post-stroke

We initially assessed the association of the baseline TyG index as continuous (Supplementary Table 2) and quartile (Supplementary Table 3) variables with myocardial injury post-stroke, respectively. The incidence of myocardial injury post-stroke increased significantly with each unit increase of TyG index after adjusting for potential confounders (odds ratio [OR]: 2.179; 95% confidence interval [CI]: 1.119–4.315; *P* = 0.023). Similarly, when evaluating the TyG index as quartiles, as compared to the individuals with low TyG index, the adjusted OR for those with moderate, high, and very high quartiles of TyG index was 1.318 (95% CI: 0.618–2.826; *P* = 0.476), 1.623 (95% CI: 0.719–3.681,* P* = 0.245), and 3.939 (95% CI: 1.289–12.315, *P* = 0.017), respectively. These results suggested that the baseline TyG index at admission was an independent risk predictor of myocardial injury post-stroke either as continuous or quartile variables.

**Fig. 2:**
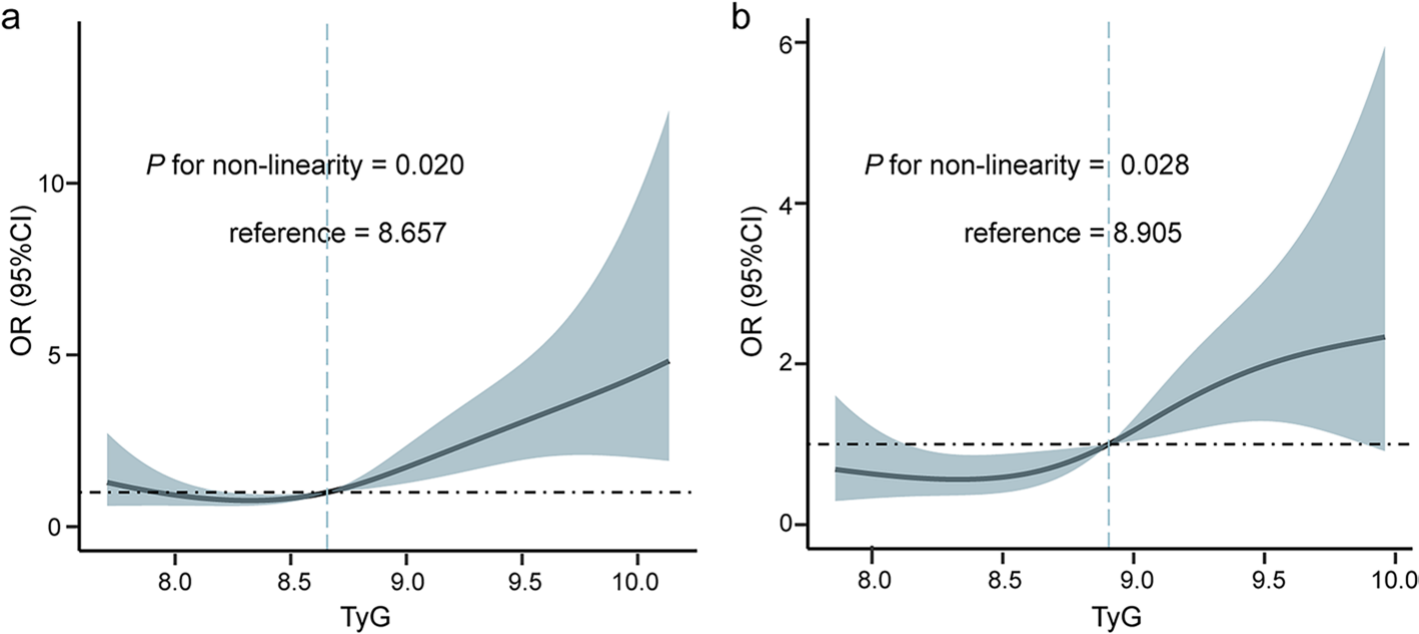
Multivariate adjusted odds ratio for myocardial injury post-stroke is based on restricted cubic spline analysis with two knots at the 25th and 75th percentiles of TyG index. Solid lines represent point estimates on the association between TyG index and myocardial injury post-stroke, and the dashed lines indicate 95% CI estimation. OR, odds ratio; TyG index, triglyceride-glucose index

We further investigated the predictive values of TyG index as dichotomous variable and found that an increased risk of myocardial injury post-stroke was substantially associated with an elevated TyG index based on the univariate analysis (OR: 2.654; 95% CI: 1.739–4.084; *P* < 0.001; Table 2). Compared to individuals with TyG index < 8.9, those with TyG index ≥ 8.9 had more significant risks of experiencing incident myocardial injury post-stroke in all four multivariate models consistently (OR range: 2.121–2.992, *P* < 0.05 for all; Table 2). Furthermore, restricted cubic spline analysis revealed that there was an adjusted dose–dependent association between TyG index and the risk of genesis of myocardial injury post-stroke (*P* for non-linearity = 0.020; Fig. 2). These findings therefore indicate that baseline TyG index was closely relevant to myocardial injury post-stroke and could be served as an early predictor for older individuals with first-ever ischemic stroke. Also, the other independent risk factors of myocardial injury post-stroke are presented in Supplementary Table 4.

**Table 2:** Association of TyG index with myocardial injury post-stroke

Analysis method	OR	95% CI	*P* value
Logistic regression analysis (*n* = 386)			
Model 1 (crude model)^a^	2.654	1.739–4.084	< 0.001
Model 2 (demographic and previous medical covariates adjusted)^b^	2.992	1.870–4.854	< 0.001
Model 3 (stroke-related covariates adjusted)^c^	2.423	1.467–4.044	0.001
Model 4 (laboratory indicators adjusted)^d^	2.121	1.209–3.753	0.009
Model 5 (fully adjusted)^e^	2.333	1.201–4.585	0.013
Propensity score analysis			
Model PSM (*n* = 254)^f^	2.196	1.416–3.478	< 0.001

*Abbreviations*: *TyG index* Triglyceride-glucose index, *OR* Odds ratio, *CI* Confidence interval, *PSM* Propensity score matching

^a^Model 1 was an unadjusted univariate regression model

^b^Model 2 included TyG index, age, sex, BMI, SBP, DBP, smoking history, history of alcohol abuse, hypertension, diabetes mellitus, peripheral vascular disease, renal dysfunction, and medications before the admission including antihypertensive agents, lipid-lowering medications, antidiabetic agents or insulin

^c^Model 3 included TyG index, stroke laterality, stroke location, stroke severity, and thrombolysis during hospitalization

^d^Model 4 included TyG index, hemoglobin, albumin, FBG, HbA1c, TC, TG, HDL-C, LDL-C, uric acid, NLR, and PLR

^e^Model 5 was adjusted for all the potential confounders. Univariate and multivariate results are represented in Supplementary Table 4

^f^254 patients were matched (1:1) using propensity score approach. Univariate result is represented in Supplementary Table 5

### Propensity score-matched analysis

**Fig. 3:**
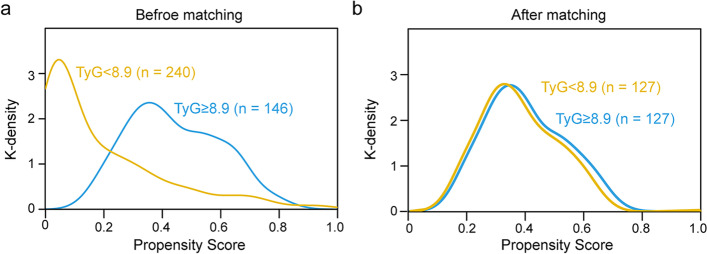
Distribution of propensity scores of patients with TyG index < 8.9 and TyG index ≥ 8.9 berofe (a) and after (b) matching. TyG index, triglyceride-glucose index

Then, we further performed PSM analysis to evaluate the association of baseline TyG index with myocardial injury post-stroke. Before propensity matching, the median propensity score in individuals with TyG index < 8.9 was 0.30 (IQR: 0.23–0.42) vs. 0.42 (IQR: 0.32–0.57) in those with TyG index ≥ 8.9. PSM yielded 127 well-matched pairs between the TyG index < 8.9 group and the TyG index ≥ 8.9 group. The distribution of propensity scores between groups before and after PSM is graphically shown in Fig. 3. After matching, propensity score distributions showed sufficient overlap. The mean (SD) propensity scores of those with TyG index < 8.9 and TyG index ≥ 8.9 were 0.40 (0.14) and 0.42 (0.15), respectively. The two groups achieved a relative balance for baseline demographic and clinical characteristics, with SMD less than 0.10 for the majority of covariates except for fasting plasma glucose, TC, TG, HDL-C, and LDL-C (Table 1). Logistic regression after PSM adjustment (n = 254) revealed that the association between baseline TyG index and myocardial injury post-stroke was still significantly robust (OR: 2.196; 95% CI: 1.416–3.478; *P* < 0.001; Table 2 and Supplementary Table 5).

### Subgroup analyses

**Fig. 4:**
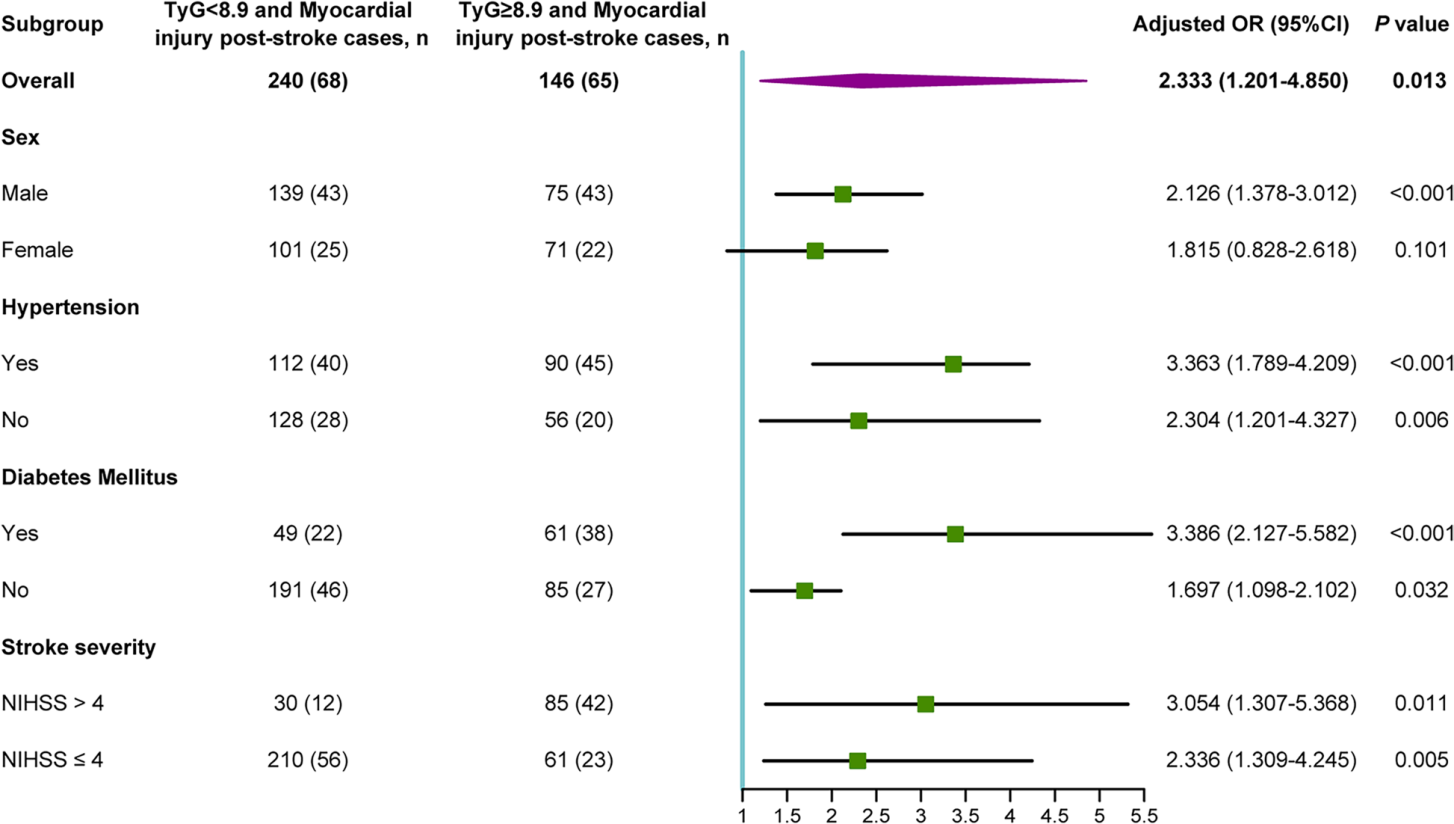
Subgroup analyses of the association of TyG index with myocardial injury post-stroke. OR, odds ratio; TyG index, triglyceride-glucose index; NIHSS, national institutes of health stroke scale

Finally, we performed subgroup analyses of the relationship between baseline TyG index and myocardial injury post-stroke according to sex, hypertension, diabetes mellitus, and stroke severity (NIHSS > 4 and NIHSS ≤ 4) (Fig. 4). Among 146 older patients with baseline TyG index ≥ 8.9, 71 (48.6%) were female, 90 (61.6%) presented with hypertension, 61 (41.8%) had diabetes mellitus, and 85 (58.2%) were with NIHSS > 4. The adjusted OR of the baseline TyG index was only significant in the male subgroup (male: OR [95% CI]: 2.126 [1.378–3.012], *P* < 0.001; female: OR [95% CI]: 1.815 [0.828–2.618], *P* = 0.101). In individuals with hypertension, there was a significant positive correlation of TyG index with myocardial injury post-stroke (OR: 3.363; 95% CI: 1.789–4.209; *P* < 0.001). The association between baseline TyG index and myocardial injury post-stroke was significant in patients with (OR: 3.386; 95% CI: 2.127–5.582; *P* < 0.001) and without (OR: 1.697; 95% CI: 1.098–2.102; *P* = 0.032) diabetes mellitus. In addition, we observed that the elevated TyG index presented a significant association with the higher risk of genesis of myocardial injury post-stroke in the NIHSS > 4 subgroup (NIHSS > 4: OR [95% CI]: 3.054 [1.307–5.368], *P* = 0.011; NIHSS ≤ 4: OR [95% CI]: 2.336 [1.309–4.245], *P* = 0.005).

## Discussion

In this cohort study of older patients with first-ever ischemic stroke who had no known preexisting cardiovascular diseases, we investigated the association between TyG index and myocardial injury post-stroke. Our results indicated that the elevated TyG index was substantially correlated with an unadjusted 2.7-fold increased risk of myocardial injury within 30 days after stroke. After adjusting for the confounding risk factors, TyG index was a significant independent predictor of myocardial injury post-stroke.

Substantial evidence has demonstrated that metabolic perturbations play essential roles in the development of various diseases [27, 28]. In particular, insulin resistance has been regarded not only as a primary or underlying cause but also as an important predictor of cardiovascular or cerebrovascular disorders [29]. At present, none of the specific methods meet the accurate and sensitive detection of insulin resistance. The gold standards to quantify insulin resistance are the euglycemic insulin clamp and intravenous glucose tolerance testing [30]. These tests are expensive, invasive, laborious, and impractical for routine clinical assessment. HOMA-IR, an approach for reflecting β-cell function decline and insulin resistance, has been widely applied in clinical practice [31]. However, HOMA-IR is not suitable for patients with insulin therapy or those without functioning β-cells [32]. To overcome the above method-inherent limitation, TyG index was exploited and proven to outperform HOMA-IR in evaluating insulin resistance in diabetic and non-diabetic patients [22]. Considering clinical effectiveness, easy availability, and appropriate cost-effectiveness, TyG index has gained tremendous amounts of attention. A recent study has revealed that a high TyG index is closely correlated with an increased risk of symptomatic coronary artery disease in patients who underwent secondary care for cardiovascular diseases [33]. Additionally, the relationship of elevated cumulative TyG index with major adverse cardiovascular events was elucidated in subjects with type 2 diabetes [34]. An increased TyG index also had improved predictive power to screen individuals at high risk for progression toward symptomatic arterial stiffness and coronary artery calcium [35].

At present, the longitudinal association between TyG index and risk of myocardial injury post-stroke remains unknown. Triglyceride and fasting plasma glucose are classical cardiometabolic indicators and routinely collected in clinical settings. TyG index was developed based on two parameters, fasting triglyceride and fasting plasma glucose to evaluate insulin resistance [22]. Our study demonstrated that the elevated TyG index exhibited a favorable predictive value for incident myocardial injury post-stroke in older patients with first-ever ischemic stroke who had no known preexisting cardiovascular disorders. The higher risk of incident myocardial injury post-stroke was still present, even after controlling for important confounders, indicating the stability and robustness of the prognostic ability. Furthermore, we found that TyG index was a potential risk indicator for identifying and predicting stroke-induced myocardial injury, particularly in male patients, hypertensive patients, diabetic individuals, and patients with neurological deficits (NIHSS > 4). These results indicated that the TyG index was substantially associated with an increased risk of stroke-induced myocardial injury. Notably, TyG index presented broader applications in stratifying individuals at high risk of myocardial injury post-stroke due to the easy availability and lower cost-effectiveness.

Myocardial injury is one of the most common complications of neurogenic cardiac injury, which is attributed to autonomic dysfunction and inflammatory response modulated by disturbance to the brain–heart axis [25]. Insulin resistance impairs glucose metabolism, which leads to hyperglycemia, local inflammatory cascade response, pro-thrombogenic effects, catecholamine release, and oxidative stress; this ultimately further aggravates coronary microvascular dysfunction and vascular endothelial damage caused by the impaired brain–heart axis [22, 36, 37]. Insulin resistance can also increase sodium retention in the kidney and activate the renin-angiotensin system to impose additional stress or injury to the myocardium [38]. Additionally, abnormal lipid metabolism may dissect the connection between insulin receptors and glucose transporters, resulting in the development of insulin resistance [39]. TC, TG, HDL-C, and LDL-C may contribute to the initiation and acceleration of atherosclerosis to promote post-stroke cardiovascular complications [40]. Thus, TyG index, including triglyceride and fasting plasma glucose, may amplify the risk of insulin resistance and atherosclerosis, thereby exacerbating stroke-induced myocardial injury, particularly in older individuals.

This cohort study has several strengths. First, no study has investigated the relationship between TyG index and the risk of stroke-induced myocardial injury in older patients with first-ever ischemic stroke who had no known preexisting cardiovascular comorbidities. Second, we attempted to apply multiple statistical approaches, such as PSM analysis, restricted cubic spline analysis, and subgroup analyses to confirm that the TyG index qualifies as a promising and practical predictor of stroke-induced myocardial injury.

This study has several inevitable limitations. First, this study is mainly limited by the single-center, retrospective design and our findings may not directly extend to other institutions. Additionally, as our sample size is relatively small, we are also aware of the fact that the statistical power is limited. Thirdly, although all stroke patients underwent history of heart disease, electrocardiogram (ECG), and transthoracic echocardiography (TTE) for cardiac evaluation, cardioembolic stroke can not be excluded. Fourth, the levels of triglyceride and fasting plasma glucose might be perturbed by the drug therapies such as lipid-lowering medications, antidiabetic agents, or insulin before hospitalization, which could be susceptible to introduce selection bias. Fifth, since HOMA-IR index is not routinely evaluated in clinical practice, additional studies on direct comparison of the predictive value of HOMA-IR and TyG index should be further assessed. Sixth, although we carefully adjusted for many potential risk factors to limit confounding, our core findings could likewise be affected by residual confounding and unmeasured risk factors. Finally, we cannot draw conclusions regarding causal relationships due to this retrospective study. Thus, larger randomized clinical trials are emergently needed to validate our findings and establish the definitive causal connection between TyG index and incident myocardial injury post-stroke.

## Conclusion

In conclusion, an elevated TyG index may be associated with a higher risk of myocardial injury post-stroke in older patients with first-ever stroke and no prior cardiovascular comorbidities, thus may serve as a complementary approach for optimized-for-risk stratification. Our findings highlight the importance of insulin resistance and the pivotal role of metabolic perturbations in neurogenic cardiac injury. Nevertheless, the potential clinical risks and benefits of TyG index-targeted treatment of myocardial injury post-stroke also require more prospective and well-designed studies to validate.

## Supplementary Information

**Additional file 1:** **Figure S1.** ROC curve of TyG index for myocardial injury post-stroke. ROC, receiver operating characteristics; TyG index, triglyceride-glucose index; AUC, area under curve. **Figure S2.** ROC curve of TyG index and other predictors for myocardial injury post-stroke. ROC, receiver operating characteristics; TyG index, triglyceride-glucose index; NIHSS, national institutes of health stroke scale. **Table S1.** Results of ROC analysis of TyG index, diabetes mellitus, hypertension, and stroke severity for predicting myocardial injury post-stroke. **Table S2.** Association between TyG index as continuous variable and myocardial injury post-stroke. **Table S3.** Association of TyG index with myocardial injury post-stroke as quartile variable. **Table S4.** Univariate and multivariate logistic regression analyses for myocardial injury post-stroke in Model 5. **Table S5.** Univariate logistic regression analysis for myocardial injurypost-stroke in the Model PSM. 

## Data Availability

The datasets generated and/or analyzed during the current study are available from the corresponding author upon reasonable request.

## Abbreviations

TyG index: Triglyceride-glucose index
PSM: Propensity score matching
OR: Odds ratio
CI: Confidence interval
HOMA-IR: Homeostasis model assessment of insulin resistance
BMI: Body mass index
SBP: Systolic blood pressure
DBP: Diastolic blood pressure
NIHSS: National institutes of health stroke scale
FBG: Fasting plasma glucose
HbA1c: Hemoglobin A1c
TC: Total cholesterol
TG: Triglyceride
HDL-C: High-density lipoprotein cholesterol
LDL-C: Low-density lipoprotein cholesterol
NLR: Neutrophil-to-lymphocyte ratio
PLR: Platelet-to-lymphocyte ratio
SD: Standard deviation
IQR: Interquartile range
ROC: Receiver operating characteristic
SMD: Standardized mean difference
