# Supplementary material for: Association of triglyceride-glucose index with myocardial injury post-stroke in older patients with first-ever ischemic stroke
Source: BMC Geriatr. 2023 Jun 8;23:357. doi: 10.1186/s12877-023-04041-7 (PMC10249284; doi:10.1186/s12877-023-04041-7)
Supplement: Supplementary file 1 — Additional file 1: Figure S1. ROC curve of TyG index for myocardial injury post-stroke. ROC, receiver operating characteristics; TyG index, triglyceride-glucose index; AUC, area under curve. Figure S2. ROC curve of TyG index and other predictors for myocardial injury post-stroke. ROC, receiver operating characteristics; TyG index, triglyceride-glucose index; NIHSS, national institutes of health stroke scale. Table S1. Results of ROC analysis of TyG index, diabetes mellitus, hypertension, and stroke severity for predicting myocardial injury post-stroke. Table S2. Association between TyG index as continuous variable and myocardial injury post-stroke. Table S3. Association of TyG index with myocardial injury post-stroke as quartile variable. Table S4. Univariate and multivariate logistic regression analyses for myocardial injury post-stroke in Model 5. Table S5. Univariate logistic regression analysis for myocardial injurypost-stroke in the Model PSM. [file 12877_2023_4041_MOESM1_ESM.doc]

SUPPLEMENTAL MATERIAL

**
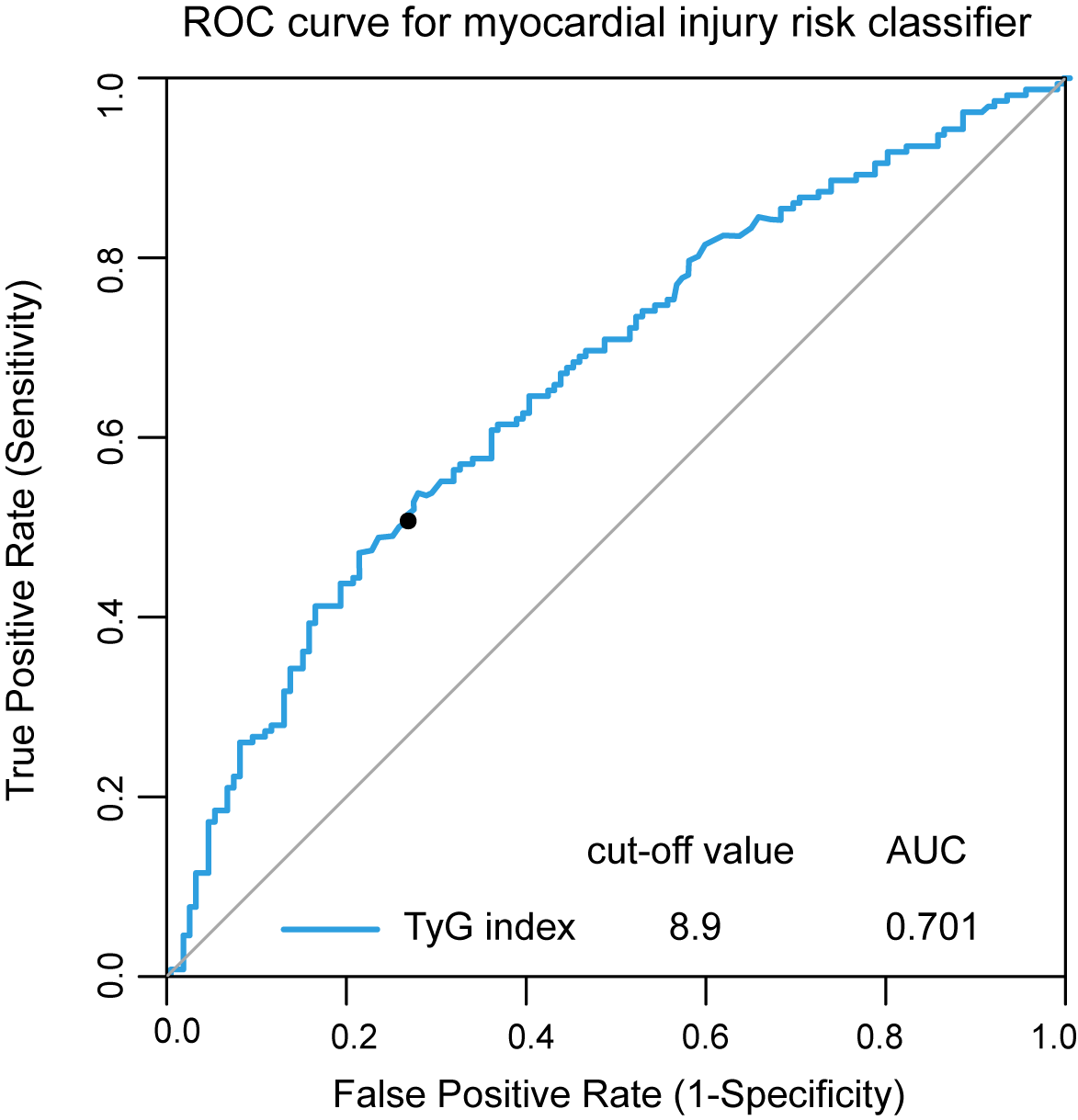
**

Figure S1. ROC curve of TyG index for myocardial injury post-stroke. ROC, receiver operating characteristics; TyG index, triglyceride-glucose index; AUC, area under curve.

**
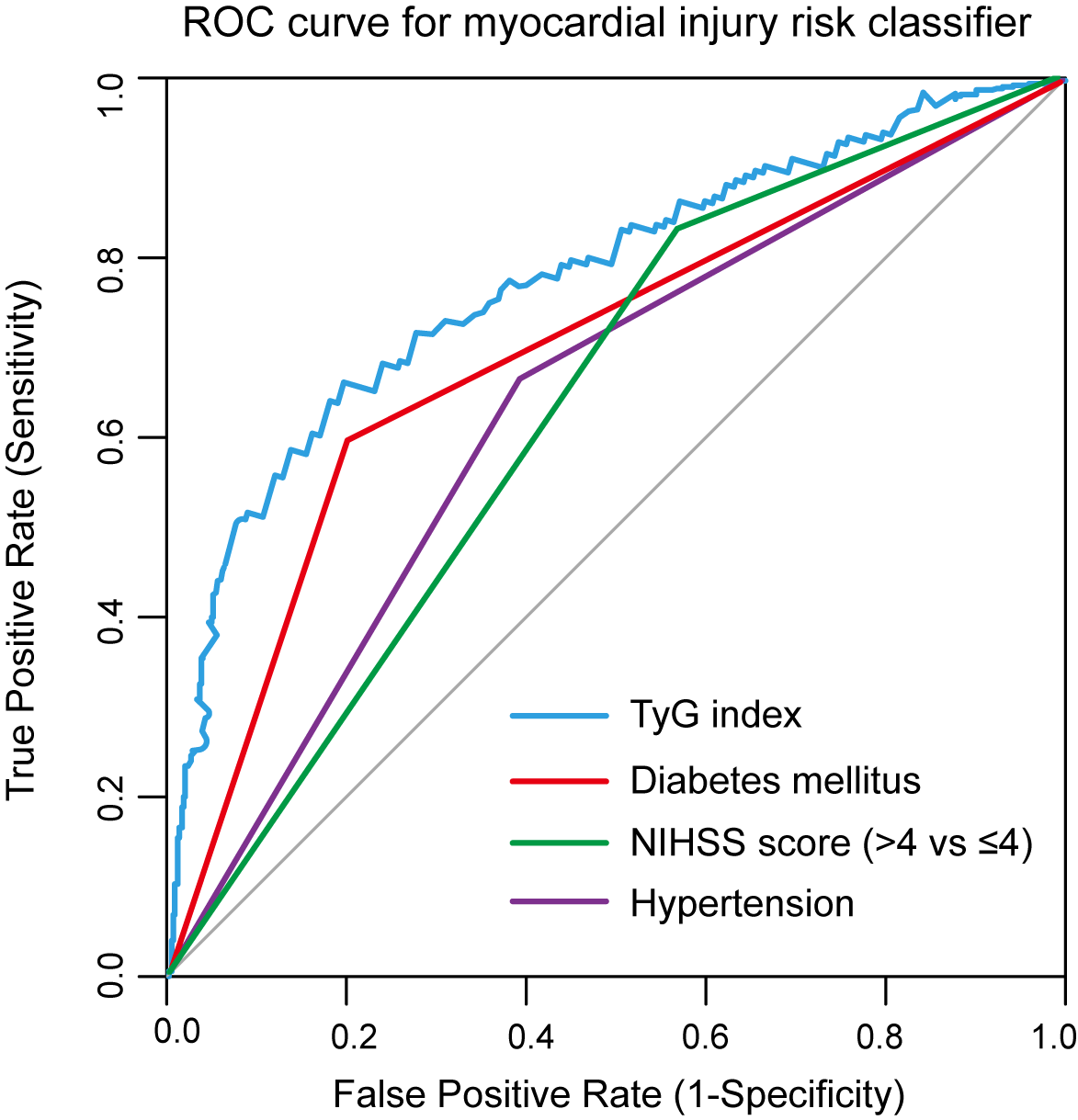
**

Figure S2. ROC curve of TyG index and other predictors for myocardial injury post-stroke. ROC, receiver operating characteristics; TyG index, triglyceride-glucose index; NIHSS, national institutes of health stroke scale.

Table S1. Results of ROC analysis of TyG index, diabetes mellitus, hypertension, and stroke severity for predicting myocardial injury post-stroke.

| Predictors | Sensitivity | Specificity | AUC | 95% CI | *P* value |
| --- | --- | --- | --- | --- | --- |
| TyG index | 0.678 | 0.755 | 0.701 | 0.673–0.758 | reference |
| Diabetes mellitus | 0.592 | 0.797 | 0.657 | 0.612–0.705 | 0.027* |
| Hypertension | 0.668 | 0.392 | 0.618 | 0.573–0.676 | 0.007* |
| Stroke severity (NIHSS > 4 vs NIHSS ≤ 4) | 0.839 | 0.573 | 0.623 | 0.587–0.693 | 0.004* |
| Abbreviations: ROC, receiver operating characteristic; TyG index, triglyceride-glucose index; AUC, area under curve; CI, confidence interval; NIHSS, national institutes of health stroke scale.  ^*^ indicates comparison with TyG index. | | | | | |

**Table S2** Association between TyG index as continuous variable and myocardial injury post-stroke.

| Variables | Univariate analysis | | Multivariate analysis | |
| --- | --- | --- | --- | --- |
|  | OR (95% CI) | *P* value | OR (95% CI) | *P* value |
| TyG index | 2.119 (1.521–2.994) | < 0.001 | 2.179 (1.119–4.315) | 0.023 |
| Age | 1.032 (1.015–1.051) | < 0.001 | 1.039 (1.013–1.066) | 0.003 |
| Sex (male vs female) | 0.763 (0.509–1.140) | 0.187 |  |  |
| BMI | 0.929 (0.876–0.982) | 0.011 | 0.940 (0.870–1.014) | 0.113 |
| SBP | 0.999 (0.988–1.010) | 0.881 |  |  |
| DBP | 0.989 (0.972–1.006) | 0.221 |  |  |
| Cigarette-smoking (Yes vs No) | 0.834 (0.544–1.278) | 0.406 |  |  |
| Alcohol (Yes vs No) | 1.275 (0.817–1.997) | 0.285 |  |  |
| Hypertension (Yes vs No) | 1.331 (1.089–1.591) | 0.026 | 1.567 (1.267–1.772) | 0.009 |
| Diabetes mellitus (Yes vs No) | 2.145 (1.367–3.403) | 0.001 | 2.034 (0.963–4.357) | 0.064 |
| Peripheral vascular disease (Yes vs No) | 0.719 (0.436–1.178) | 0.192 |  |  |
| Renal dysfunction (Yes vs No) | 1.243 (0.324–5.091) | 0.748 |  |  |
| Antihypertensive agents (Yes vs No) | 0.999 (0.670–1.490) | 0.996 |  |  |
| Lipid-lowering agents (Yes vs No) | 1.105 (0.573–2.144) | 0.765 |  |  |
| Antidiabetic agents or insulin (Yes vs No) | 1.971 (1.290–3.033) | 0.002 | 1.163 (0.588–2.291) | 0.663 |
| Stroke laterality |  |  |  |  |
| Left | reference |  | reference |  |
| Right | 1.056 (0.663–1.685) | 0.818 | 0.925 (0.519–1.645) | 0.790 |
| Bilateral | 1.736 (1.041–2.917) | 0.036 | 1.637 (0.832–3.251) | 0.155 |
| Stroke location |  |  |  |  |
| Cortical | reference |  | reference |  |
| Subcortical | 0.387 (0.213–0.688) | 0.001 | 0.184 (0.083–0.394) | < 0.001 |
| Cerebellar | 0.500 (0.086–2.893) | 0.419 | 0.095 (0.009–0.874) | 0.041 |
| Brainstem | 0.500 (0.057–4.392) | 0.502 | 0.125 (0.009–1.491) | 0.092 |
| Multiple | 0.546 (0.287–1.019) | 0.060 | 0.241 (0.103–0.546) | 0.001 |
| Stroke severity (NIHSS) | 1.101 (1.031–1.180) | 0.005 | 0.958 (0.858–1.070) | 0.445 |
| Thrombolysis (Yes vs No) | 1.495 (0.722–3.186) | 0.284 |  |  |
| Hemoglobin | 0.988 (0.978–1.108) | 0.566 |  |  |
| Albumin | 0.897 (0.851–0.943) | < 0.001 | 0.902 (0.838–0.967) | 0.004 |
| NLR | 1.073 (1.015–1.144) | 0.021 | 1.035 (0.941–1.148) | 0.501 |
| PLR | 0.933 (1.001–1.006) | 0.275 |  |  |
| HbA1c | 1.254 (1.008–1.477) | 0.025 | 0.975 (0.750–1.276) | 0.852 |
| FBG | 1.153 (1.048–1.278) | 0.005 | 1.372 (1.038–1.818) | 0.002 |
| TC | 1.561 (1.277–1.925) | < 0.001 | 0.780 (0.398–1.494) | 0.396 |
| TG | 1.467 (1.158–1.886) | 0.002 | 1.678 (1.233–2.012) | 0.001 |
| HDL-C | 0.113 (0.053–0.233) | < 0.001 | 0.556 (0.012–1.125) | 0.239 |
| LDL-C | 1.883 (1.466–2.452) | < 0.001 | 2.411 (1.137–5.390) | 0.026 |
| Uric acid | 0.999 (0.996–1.001) | 0.211 |  |  |
| Abbreviations: TyG index, triglyceride-glucose index; OR, odds ratio; CI, confidence interval; BMI, body mass index; SBP, systolic blood pressure; DBP, diastolic blood pressure; NIHSS, national institutes of health stroke scale; NLR, neutrophil-to-lymphocyte ratio; PLR, platelet-to-lymphocyte ratio; FBG: fasting plasma glucose; HbA1c, hemoglobin A1c; TC, total cholesterol; TG, triglyceride; HDL-C, high-density lipoprotein cholesterol; LDL-C; low-density lipoprotein cholesterol. | | | | |

**Table S3** Association of TyG index with myocardial injury post-stroke as quartile variable.

| Variables | Univariate analysis | | Multivariate analysis | |
| --- | --- | --- | --- | --- |
|  | OR (95% CI) | *P* value | OR (95% CI) | *P* value |
| TyG index |  |  |  |  |
| Low (7.35–8.30) | reference |  | reference |  |
| Moderate (8.30–8.66) | 0.974 (0.547–1.735) | 0.930 | 1.318 (0.618–2.826) | 0.476 |
| High (8.66–9.18) | 1.487 (0.843–2.639) | 0.172 | 1.623 (0.719–3.681) | 0.245 |
| Very high (9.18–10.80) | 3.665 (2.033–6.739) | < 0.001 | 3.939 (1.289–12.315) | 0.017 |
| Age | 1.032 (1.015–1.051) | < 0.001 | 1.037 (1.011–1.064) | 0.005 |
| Sex (male vs female) | 0.763 (0.509–1.140) | 0.187 |  |  |
| BMI | 0.929 (0.876–0.982) | 0.011 | 0.937 (0.866–1.012) | 0.098 |
| SBP | 0.999 (0.988–1.010) | 0.881 |  |  |
| DBP | 0.989 (0.972–1.006) | 0.221 |  |  |
| Cigarette-smoking (Yes vs No) | 0.834 (0.544–1.278) | 0.406 |  |  |
| Alcohol (Yes vs No) | 1.275 (0.817–1.997) | 0.285 |  |  |
| Hypertension (Yes vs No) | 1.331 (1.089–1.591) | 0.026 | 0.603 (0.214–1.146) | 0.189 |
| Diabetes mellitus (Yes vs No) | 2.145 (1.367–3.403) | 0.001 | 2.085 (1.288–4.458) | 0.015 |
| Peripheral vascular disease (Yes vs No) | 0.719 (0.436–1.178) | 0.192 |  |  |
| Renal dysfunction (Yes vs No) | 1.243 (0.324–5.091) | 0.748 |  |  |
| Antihypertensive agents (Yes vs No) | 0.999 (0.670–1.490) | 0.996 |  |  |
| Lipid-lowering agents (Yes vs No) | 1.105 (0.573–2.144) | 0.765 |  |  |
| Antidiabetic agents or insulin (Yes vs No) | 1.971 (1.290–3.033) | 0.002 | 1.190 (0.600–2.354) | 0.616 |
| Stroke laterality |  |  |  |  |
| Left | reference |  | reference |  |
| Right | 1.056 (0.663–1.685) | 0.818 | 0.931 (0.522–1.656) | 0.806 |
| Bilateral | 1.736 (1.041–2.917) | 0.036 | 1.579 (0.797–3.159) | 0.192 |
| Stroke location |  |  |  |  |
| Cortical | reference |  | reference |  |
| Subcortical | 0.387 (0.213–0.688) | 0.001 | 0.180 (0.081–0.384) | < 0.001 |
| Cerebellar | 0.500 (0.086–2.893) | 0.419 | 0.120 (0.011–1.088) | 0.063 |
| Brainstem | 0.500 (0.057–4.392) | 0.502 | 0.101 (0.007–1.267) | 0.071 |
| Multiple | 0.546 (0.287–1.019) | 0.060 | 0.244 (0.104–0.553) | 0.001 |
| Stroke severity (NIHSS) | 1.101 (1.031–1.180) | 0.005 | 0.954 (0.853–1.066) | 0.404 |
| Thrombolysis (Yes vs No) | 1.495 (0.722–3.186) | 0.284 |  |  |
| Hemoglobin | 0.988 (0.978–1.108) | 0.566 |  |  |
| Albumin | 0.897 (0.851–0.943) | < 0.001 | 0.903 (0.840–0.968) | 0.005 |
| NLR | 1.073 (1.015–1.144) | 0.021 | 1.038 (0.943–1.152) | 0.464 |
| PLR | 0.933 (1.001–1.006) | 0.275 |  |  |
| HbA1c | 1.254 (1.008–1.477) | 0.025 | 0.994 (0.772–1.290) | 0.966 |
| FBG | 1.153 (1.048–1.278) | 0.005 | 1.372 (1.038–1.818) | 0.027 |
| TC | 1.561 (1.277–1.925) | < 0.001 | 0.772 (0.391–1.485) | 0.444 |
| TG | 1.467 (1.158–1.886) | 0.002 | 1.789 (1.201–1.890) | 0.018 |
| HDL-C | 0.113 (0.053–0.233) | < 0.001 | 0.356 (0.036–1.456) | 0.193 |
| LDL-C | 1.883 (1.466–2.452) | < 0.001 | 1.001 (1.120–5.437) | 0.029 |
| Uric acid | 0.999 (0.996–1.001) | 0.211 |  |  |
| Abbreviations: TyG index, triglyceride-glucose index; OR, odds ratio; CI, confidence interval; BMI, body mass index; SBP, systolic blood pressure; DBP, diastolic blood pressure; NIHSS, national institutes of health stroke scale; NLR, neutrophil-to-lymphocyte ratio; PLR, platelet-to-lymphocyte ratio; FBG: fasting plasma glucose; HbA1c, hemoglobin A1c; TC, total cholesterol; TG, triglyceride; HDL-C, high-density lipoprotein cholesterol; LDL-C; low-density lipoprotein cholesterol. | | | | |

**Table S4** Univariate and multivariate logistic regression analyses for myocardial injury post-stroke in Model 5.

| Variables | Univariate analysis | | Multivariate analysis | |
| --- | --- | --- | --- | --- |
|  | OR (95% CI) | *P* value | OR (95% CI) | *P* value |
| TyG index (< 8.9 vs ≥ 8.9) | 2.654 (1.739–4.084) | < 0.001 | 2.333 (1.201–4.850) | 0.013 |
| Age | 1.032 (1.015–1.051) | < 0.001 | 1.037 (1.012–1.064) | 0.004 |
| Sex (male vs female) | 0.763 (0.509–1.140) | 0.187 |  |  |
| BMI | 0.929 (0.876–0.982) | 0.011 |  |  |
| SBP | 0.999 (0.988–1.010) | 0.881 |  |  |
| DBP | 0.989 (0.972–1.006) | 0.221 |  |  |
| Cigarette-smoking (Yes vs No) | 0.834 (0.544–1.278) | 0.406 |  |  |
| Alcohol (Yes vs No) | 1.275 (0.817–1.997) | 0.285 |  |  |
| Hypertension (Yes vs No) | 1.331 (1.089–1.591) | 0.026 | 1.170 (1.269–1.690) | 0.032 |
| Diabetes mellitus (Yes vs No) | 2.145 (1.367–3.403) | 0.001 | 2.143 (1.020–4.568) | 0.046 |
| Peripheral vascular disease (Yes vs No) | 0.719 (0.436–1.178) | 0.192 |  |  |
| Renal dysfunction (Yes vs No) | 1.243 (0.324–5.091) | 0.748 |  |  |
| Antihypertensive agents (Yes vs No) | 0.999 (0.670–1.490) | 0.996 |  |  |
| Lipid-lowering agents (Yes vs No) | 1.105 (0.573–2.144) | 0.765 |  |  |
| Antidiabetic agents or insulin (Yes vs No) | 1.971 (1.290–3.033) | 0.002 | 1.169 (0.591–2.304) | 0.651 |
| Stroke laterality |  |  |  |  |
| Left | reference |  | reference |  |
| Right | 1.056 (0.663–1.685) | 0.818 | 0.900 (0.506–1.599) | 0.719 |
| Bilateral | 1.736 (1.041–2.917) | 0.036 | 1.579 (0.802–3.135) | 0.188 |
| Stroke location |  |  |  |  |
| Cortical | reference |  | reference |  |
| Subcortical | 0.387 (0.213–0.688) | 0.001 | 0.183 (0.083–0.389) | < 0.001 |
| Cerebellar | 0.500 (0.086–2.893) | 0.419 | 0.095 (0.008–0.948) | 0.050 |
| Brainstem | 0.500 (0.057–4.392) | 0.502 | 0.128 (0.010–1.548) | 0.100 |
| Multiple | 0.546 (0.287–1.019) | 0.060 | 0.242 (0.104–0.547) | 0.001 |
| Stroke severity (NIHSS) | 1.101 (1.031–1.180) | 0.005 | 0.978 (0.883–1.083) | 0.670 |
| Thrombolysis (Yes vs No) | 1.495 (0.722–3.186) | 0.284 |  |  |
| Hemoglobin | 0.988 (0.978–1.108) | 0.566 |  |  |
| Albumin | 0.897 (0.851–0.943) | < 0.001 | 0.898 (0.834–0.962) | 0.003 |
| NLR | 1.073 (1.015–1.144) | 0.021 | 1.034 (0.938–1.148) | 0.523 |
| PLR | 0.933 (1.001–1.006) | 0.275 |  |  |
| HbA1c | 1.254 (1.008–1.477) | 0.025 | 1.221 (1.118–1.313) | 0.029 |
| FBG | 1.153 (1.048–1.278) | 0.005 | 1.398 (1.194–1.621) | 0.006 |
| TC | 1.561 (1.277–1.925) | < 0.001 | 1.395 (1.056–1.848) | 0.033 |
| TG | 1.467 (1.158–1.886) | 0.002 | 1.960 (1.519–2.102) | 0.015 |
| HDL-C | 0.113 (0.053–0.233) | < 0.001 | 0.370 (0.206–0.534) | 0.026 |
| LDL-C | 1.883 (1.466–2.452) | < 0.001 | 1.467 (1.233–1.999) | 0.012 |
| Uric acid | 0.999 (0.996–1.001) | 0.211 |  |  |
| Abbreviations: TyG index, triglyceride-glucose index; OR, odds ratio; CI, confidence interval; BMI, body mass index; SBP, systolic blood pressure; DBP, diastolic blood pressure; NIHSS, national institutes of health stroke scale; NLR, neutrophil-to-lymphocyte ratio; PLR, platelet-to-lymphocyte ratio; FBG: fasting plasma glucose; HbA1c, hemoglobin A1c; TC, total cholesterol; TG, triglyceride; HDL-C, high-density lipoprotein cholesterol; LDL-C; low-density lipoprotein cholesterol. | | | | |

**Table S5** Univariate logistic regression analysis for myocardial injury post-stroke in the Model PSM.

| Variables | Univariate analysis | |
| --- | --- | --- |
|  | OR (95% CI) | *P* value |
| TyG index (< 8.9 vs ≥ 8.9) | 2.196 (1.416–3.478) | <0.001 |
| Age | 1.052 (0.827–1.080) | 0.432 |
| Sex (male vs female) | 1.322 (0.805–2.180) | 0.271 |
| BMI | 0.882 (0.813–1.153) | 0.232 |
| SBP | 0.998 (0.984–1.011) | 0.732 |
| DBP | 0.977 (0.956–0.998) | 0.037 |
| Cigarette-smoking (Yes vs No) | 0.753 (0.447–1.263) | 0.282 |
| Alcohol (Yes vs No) | 0.910 (0.528–1.570) | 0.734 |
| Hypertension (Yes vs No) | 0.601 (0.359–1.998) | 0.450 |
| Diabetes mellitus (Yes vs No) | 1.480 (0.876–2.519) | 0.145 |
| Peripheral vascular disease (Yes vs No) | 0.682 (0.382–1.211) | 0.193 |
| Renal dysfunction (Yes vs No) | 1.373 (0.224–10.559) | 0.731 |
| Antihypertensive agents (Yes vs No) | 0.873 (0.528–1.442) | 0.597 |
| Lipid-lowering agents (Yes vs No) | 0.898 (0.416–1.937) | 0.783 |
| Antidiabetic agents or insulin (Yes vs No) | 1.483 (0.898–2.461) | 0.125 |
| Stroke laterality |  |  |
| Left | reference |  |
| Right | 1.002 (0.559–1.790) | 0.998 |
| Bilateral | 1.400 (0.749–2.635) | 0.294 |
| Stroke location |  |  |
| Cortical | reference |  |
| Subcortical | 0.319 (0.142–0.674) | 0.004 |
| Cerebellar | 0.122 (0.006–1.067) | 0.082 |
| Brainstem | 0.333 (0.234–1.112) | 0.986 |
| Multiple | 0.416 (0.178–0.926) | 0.036 |
| Stroke severity (NIHSS) | 1.102 (1.013–1.203) | 0.026 |
| Thrombolysis (Yes vs No) | 1.234 (0.503–3.130) | 0.647 |
| Hemoglobin | 0.979 (0.966–0.992) | 0.002 |
| Albumin | 0.882 (0.826–0.938) | < 0.001 |
| NLR | 0.806 (0.626–1.211) | 0.218 |
| PLR | 1.005 (1.001–1.009) | 0.009 |
| HbA1c | 1.239 (0.836–1.507) | 0.129 |
| FBG | 1.145 (1.022–1.294) | 0.024 |
| TC | 1.742 (1.360–2.266) | < 0.001 |
| TG | 1.266 (0.957–1.700) | 0.105 |
| HDL-C | 0.130 (0.051–0.310) | < 0.001 |
| LDL-C | 2.336 (1.695–3.307) | <0.001 |
| Uric acid | 0.996 (0.994–0.109) | 0.887 |
| Abbreviations: TyG index, triglyceride-glucose index; OR, odds ratio; CI, confidence interval; PSM, propensity score matching; BMI, body mass index; SBP, systolic blood pressure; DBP, diastolic blood pressure; NIHSS, national institutes of health stroke scale; NLR, neutrophil-to-lymphocyte ratio; PLR, platelet-to-lymphocyte ratio; FBG: fasting plasma glucose; HbA1c, hemoglobin A1c; TC, total cholesterol; TG, triglyceride; HDL-C, high-density lipoprotein cholesterol; LDL-C; low-density lipoprotein cholesterol. | | |
